# Biogenic nanoporous silicon carrier improves the efficacy of buparvaquone against resistant visceral leishmaniasis

**DOI:** 10.1371/journal.pntd.0009533

**Published:** 2021-06-29

**Authors:** Rinez Thapa, Subhasish Mondal, Joakim Riikonen, Jimi Rantanen, Simo Näkki, Tuomo Nissinen, Ale Närvänen, Vesa-Pekka Lehto

**Affiliations:** 1 Department of Applied Physics, University of Eastern Finland, Kuopio, Finland; 2 School of Pharmacy, The Neotia University, Sarisa, West Bengal, India; 3 School of Pharmacy, University of Eastern Finland, Kuopio, Finland; London School of Hygiene and Tropical Medicine, UNITED KINGDOM

## Abstract

Visceral leishmaniasis is a vector-borne protozoan infection that is fatal if untreated. There is no vaccination against the disease, and the current chemotherapeutic agents are ineffective due to increased resistance and severe side effects. Buparvaquone is a potential drug against the leishmaniases, but it is highly hydrophobic resulting in poor bioavailability and low therapeutic efficacy. Herein, we loaded the drug into silicon nanoparticles produced from barley husk, which is an agricultural residue and widely available. The buparvaquone-loaded nanoparticles were several times more selective to kill the intracellular parasites being non-toxic to macrophages compared to the pure buparvaquone and other conventionally used anti-leishmanial agents. Furthermore, the *in vivo* results revealed that the intraperitoneally injected buparvaquone-loaded nanoparticles suppressed the parasite burden close to 100%. By contrast, pure buparvaquone suppressed the burden only by 50% with corresponding doses. As the conclusion, the biogenic silicon nanoparticles are promising carriers to significantly improve the therapeutic efficacy and selectivity of buparvaquone against resistant visceral leishmaniasis opening a new avenue for low-cost treatment against this neglected tropical disease threatening especially the poor people in developing nations.

## Author summary

Visceral leishmaniasis is a neglected tropical disease caused by a protozoan parasite. It infects macrophage associated organs and invades the immune system. Currently, there is no vaccination against the disease and the conventional chemotherapeutic agents are ineffective due to the increased resistance and toxicity. In the present work, we utilized porous silicon nanoparticles as carriers for an anti-leishmanial drug (buparvaquone) to improve its therapeutic efficacy against visceral leishmaniasis (S1 Scheme). The nanoparticles were synthesized from an agricultural residue (barley husk), which is cheap and widely available. Due to the nano size of the nanoparticles, they internalized into the infected cells successfully. The efficacies of buparvaquone loaded in the nanoparticles were greatly improved in comparison with pure buparvaquone to evacuate the parasitic infection both *in vitro* and *in vivo* studies. Therefore, the biogenic silicon nanocarriers are promising candidates to be utilized as low-cost treatment regimen in leishmaniases therapy.

## Introduction

Visceral leishmaniasis (VL) is a deadly disease with a mortality rate of over 95% if untreated [1]. It invades the immune system and damages the liver, spleen, bone marrow, and lymph nodes [2]. There is no vaccination against the disease, and every year 50 000 to 90 000 new cases are reported worldwide [1].

VL is protozoan infection caused by *L*. *donovani* and *L*. *infantum*, which is transmitted by the bite of infected sandflies of genus *Phlebotomus* in the old world and *Lutzomya* in the new world [2]. In the gut of the sandfly, the parasite exists in promastigote forms, which are motile, extracellular, and flagellated. Human infection takes place when the promastigotes are ingested by the macrophages and metamorphosed into nonmotile amastigote forms [3]. The amastigotes multiply by binary fission until the macrophage eventually bursts. Subsequently, the amastigotes are released into the bloodstream, and they infect all organs containing macrophages and the reticuloendothelial system, where they are hosted for their replication [3].

Current therapy against VL solely relies on chemotherapy, in which the drugs are expensive, highly toxic, difficult to administer, and impose side effects [2–6]. Moreover, conventional drugs are becoming ineffective due to the increasing drug resistance. For instance, antimonials like sodium stibogluconate (SSG) have been used as the standard first line treatment for the past several decades, but it is no longer practical due to the prevailing resistance by the parasites. Thus, high dose regimen is prescribed [4,5]. Due to the high dose, severe side effects, including cardiac arrhythmia and acute pancreatitis, have been reported [5]. Similarly, other pharmaceuticals such as amphotericin B (AmB), paromomycin (PMM) and miltefosine (MF) used in the chemotherapy are either expensive, suffer from resistance, or induce severe side effects associated with fever, bone pain, kidney failure, anemia, ototoxicity and gastrointestinal toxicity [2,4–6]. To reduce the side effects, liposomal formulation of AmB (AmBisome) have been developed but its wider utilization is limited due to the high cost (2800 US$/treatment), which is critical issue especially for the poor people in the endemic regions in the developing countries [5,6]. Therefore, novel affordable approaches using potential therapeutics are needed to inhibit the growth of *L*. *donovani* amastigotes.

**Fig 1:**
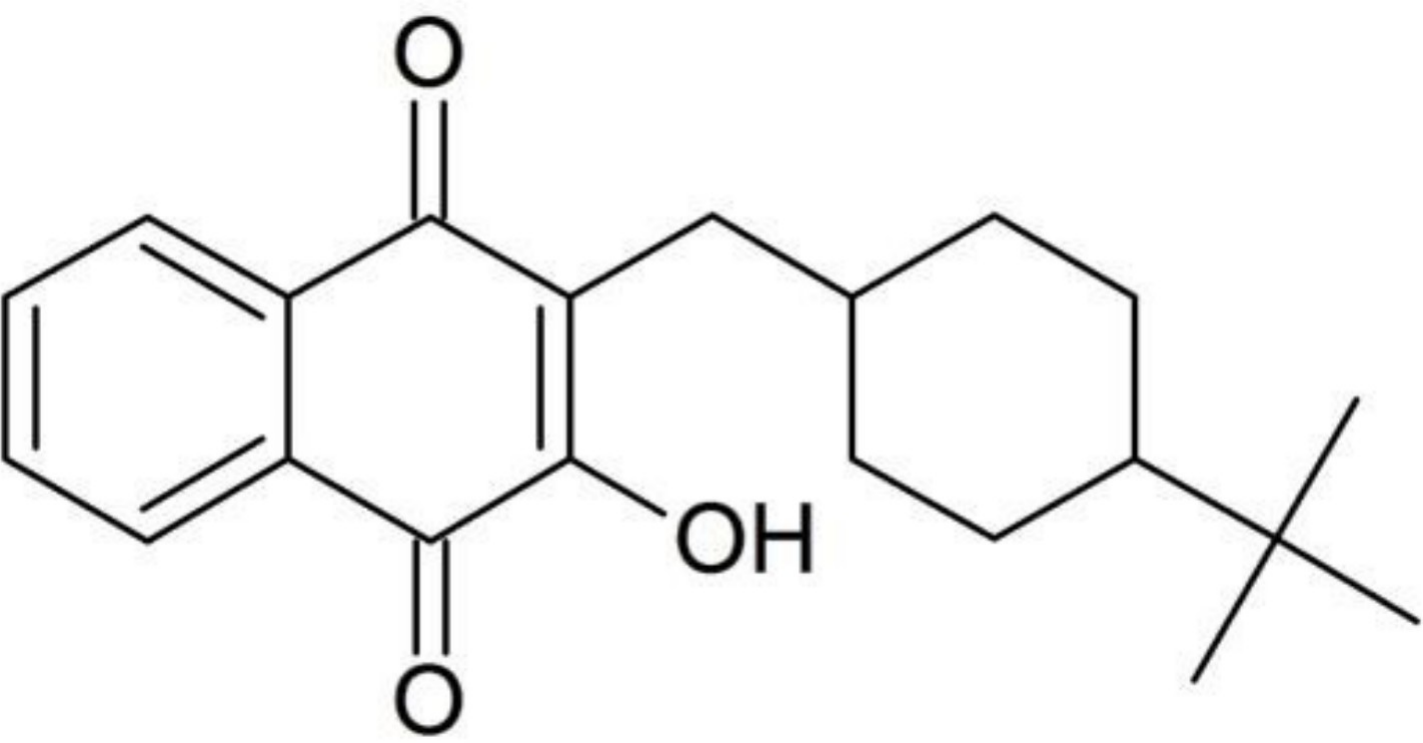
Chemical structure of buparvaquone.

Naphthoquinones are widely distributed organic compounds in nature that exhibit antibacterial, anti-inflammatory, and antipyretic properties [7]. Buparvaquone (BPQ) (Fig 1), market as Butalex, a member of hydroxynaphthoquinones, is a safe drug for veterinary treatment and has been the only effective commercial treatment against bovine theileriosis since the late 1980s [8–10]. In 1992, Croft *et al*., demonstrated the excellent *in vitro* activity of BPQ against *L*. *donovani* amastigotes, but its efficacy *in vivo* was limited by the poor bioavailability due to very low aqueous solubility (<0.03 μg/mL) [11,12].

In the present study, we employed nanoporous silicon (PSi) as carriers for BPQ to improve its therapeutic efficacy against VL. The main advantages of using PSi nanocarriers are associated with their biocompatibility and high drug loading capacity facilitated by the large surface area and the porous structure. Herein, PSi was produced following the circular economy principle utilizing barley husk as the raw material, which is a widely available low-cost agricultural residue. Further, the PSi surfaces were stabilized with thermal hydrocarbonization which after carboxylic acid functional groups were added [13,14]. The hydrocarbonized surfaces were preferred to enhance the loading of BPQ through hydrophobic interaction, and the further functionalization was made to improve the colloidal stability of the nanoparticles. The *in vitro* and *in vivo* antileishmanial efficacies of the BPQ loaded nanoparticles were examined and compared with the pure BPQ, SSG, AmB, PMM, and MF against the wild type as well as resistant strains of *L*. *donovani*.

## Methods

### Ethics statements

BALB/c mice weighing between 20–22 g (female, 6–8 weeks old) were used for the study. The experimental protocols were approved by the University Animal Ethics Committee, and procedures were in accordance with the NIH guidelines (National Institutes of Health guide for the care and use of Laboratory animals, NIH Publications No. 8023, revised 1978). Mice were housed five per cage and maintained in standard pathogen-free environmental conditions (12: 12 h light: dark cycle at 22 ± 2°C) with access to food and water *ad libitum*.

### Reagents

Barley husk ash (donated by Altia Oyj), hydrofluoric acid (HF 38–40%, Merck), ethanol (EtOH 99.5%, Altia Oyj), hydrochloric acid (HCl 37–39%, Merck), sodium chloride (NaCl, Fisher Scientific UK), undecylenic acid (Merck), chloroform (Sigma-Aldrich), and Tween 20 (Sigma-Aldrich Chemie GmbH) were used in the preparation of the nanoparticles. Mannitol (Merck) was used as an excipient in the freeze-drying of the nanoparticles. Sodium acetate (NaOAc, Merck) and HPLC grade methanol (MeOH, VWR chemicals, Prolabo) were used in preparation of the mobile phase solution in HPLC analysis. Buparvaquone (BPQ) was prepared as described previously [12]. Amphotericin B (AmB), paromomycin (PMM), miltefosine (MF), medium 199, RPMI-1640, and fetal calf serum were purchased from Sigma Aldrich (USA). Sodium stibogluconate (SSG) was a generous gift from Albert David Ltd. (Kolkata, India). Dulbecco’s Modified Eagle’s Medium (DMEM, ATCC 30–2002) and Fetal Bovine Serum (FBS, GE Healthcare Life Sciences) were used in cell culture.

### HPLC setup and conditions

HPLC (Shimadzu, Japan) was used to measure the loading degree of BPQ and study the release kinetics in aqueous solutions. The HPLC system consisted of an LC–10 AT VP pump, a SIL-10 AD VP sample injector and an FCV-10 AL UV/vis detector. The HPLC analysis was carried at room temperature using Inertsil ODS-3 C_18_, 4×150 mm (GL Sciences Inc.) analytical column. The mobile solution was 50 mM NaOAc and 85% MeOH at pH 3.4. The samples were diluted with a mobile solution before running. The flow rate was 1 mL/min, injection volume was 0.1 mL, and the analysis time was 20 min per sample. The samples were monitored at 262 nm. The BPQ concentrations were determined by measuring the peak areas compared to the calibration curves of BPQ standards.

### Synthesis of the nanoparticles (NP)

Barley husk ash (32 g) was washed in 375 mL aqueous solution of 10 wt.% HCl in 100°C water bath for 2 h to remove inorganic impurities. The washed powder was then filtered out and rinsed with copious amounts of water and dried at 120°C for 2 h. The sample was then calcined at 600°C in air for 3 h to remove organic impurities. The purification procedure was repeated once and altogether 24 g of purified porous SiO_2_ was obtained.

Nanostructured PSi was produced from the purified SiO_2_ powder using magnesiothermic reduction [15]. Briefly, 4 g of silica powder was mixed with 3.24 g of Mg and 4.0 g of NaCl, and milled in a planetary ball mill (Fritsch Pulverisette 7 Premium line) using 1 mm zirconia balls at 400 rpm for 4 min. The milled powder was loaded into a quartz tube and placed into a tube oven at 300°C in a nitrogen (N_2_) atmosphere. The sample was then slowly heated up to 600°C with a heating rate of 5°C/min and kept at 600°C for 4 h. The magnesiothermic reduction procedure was repeated four times and the products were pooled together. After reduction, the side products (MgO, Mg_2_Si, Mg) were dissolved first in 400 mL of 37% HCl at 70°C for 2 h followed by washing with water. Remaining SiO_2_ was removed by washing in 1:1 HF:EtOH solution in an ice bath for 5 min. The PSi powder was then filtered out and dried at 65°C for 1 h yielding 2.8 g of PSi particles.

Nanoparticles were prepared by further milling the PSi powder in 18 ml of EtOH at 800 rpm for 140 min at 5 min intervals. After milling, the nanoparticles were washed with the 1:1 HF:EtOH solution to remove SiO_2_ produced during the milling. The particles were separated from the solution by centrifugation and dried at 65°C for 1 h.

Thermal hydrocarbonization of PSi (THCPSi) was performed through reaction with acetylene at elevated temperature. First, the PSi powder (0.95 g) was flushed with 1 L/min N_2_ gas for 30 min using a quartz tube. Acetylene gas (1 L/min) was introduced into the tube while maintaining the N_2_ flow. After 15 min, the tube was placed in a tube oven preheated to 500°C for 14 min 30 s. The acetylene flow was stopped and after 30 s in N_2_ flow at 500°C, the tube was removed from the tube oven and let to cool down to room temperature in the N_2_ flow.

Carboxylic acid (-COOH) functional groups were grafted on the THCPSi surfaces by following the method described previously [14,16]. Briefly, THCPSi was mixed with 30 mL of undecylenic acid into the quartz tube. The sample was placed in an oven and incubated at 120°C for 16 h while maintaining the nitrogen flow. The undecylenic acid functionalized THCPSi was washed with chloroform on a filter and dried at 65°C for 1 h. The particles were then redispersed by sonicating in 1% Tween solution for 5.5 h, and separated from the suspension by centrifugation at 7000 g for 10 min. The nanoparticles were then washed with EtOH to remove Tween, and finally, the medium was changed to deionized water. The synthesized UnTHCPSi nanoparticles are denoted as NP.

### BPQ loading/release study and freeze-drying of the nanoparticles

NP (20 mg) was washed with 20 mL EtOH using centrifugation (20 min at 13200 rpm, Eppendorf Centrifuge 5424, Eppendorf AG). After the washing, NP was mixed with 20 mg BPQ suspended in 20 mL EtOH and incubated for 2 h at room temperature. NP was centrifuged, and the BPQ concentrations in the supernatant were measured with HPLC before and after mixing. The loading degree (% w/w) of BPQ in the nanoparticles (BPQ+NP) was calculated using following equation

$$\%\;w/w=\frac{m_{d}}{m_{d}+m_{NP}}\times 100$$ (1)

where m_d_ is the mass of loaded BPQ and m_NP_ represents the mass of UnTHCPSi nanoparticles. The m_d_ was calculated from the BPQ concentrations in the supernatant, before and after the loading.

For the long-term storage, the nanoparticles were freeze-dried using mannitol, which is a typical excipient among pharmaceuticals [17]. The aliquots of nanoparticles (1 mg/mL) in aqueous solution of 4 wt.% mannitol, was sonicated for 5 minutes, kept in a freezer (-40°C) for 45 min and then freeze-dried for 48 h through lyophilization (Edwards Lyophilizator). The freeze-dried cakes of the nanoparticles were stored at 4°C.

The freeze-dried nanoparticles were used to test the release kinetics of the BPQ. The experiment was done in EtOH, Milli-Q water, and cell culture medium (DMEM). For each release medium, 0.5 mg of the BPQ+NP was redispersed in 1 mL of the respective medium and incubated at room temperature. At pre-defined time points (0 h, 0.5 h, 1 h, 2 h, 4 h, and 24 h), NP was centrifuged, and 500 μL of release medium was taken. Fresh 500 μL of the corresponding medium was added to the NP and incubated further until the next time point. The EtOH and water samples were evaporated and dissolved with MeOH. The DMEM samples were filtrated with Amicon Ultrafree-Cl filters cut off 10 000 (Millipore), evaporated, and then dissolved with MeOH. The released BPQ concentrations were measured with HPLC as described above.

### Characterization of the nanoparticles

The crystallite size of NP was determined with an X-ray diffractometer (XRD, Bruker D8 DISCOVER) equipped with a Cu-anode X-ray tube (λ = 1.54 Å). Freshly synthesized NP was dried at 65°C overnight, and the measurements were done on a 2θ range of 20° - 80° and a step size of 0.031°. The measurement time was 9 min 32 s. The X-rays were limited on the primary side with a motorized slit set to illuminate 2 cm length on the sample, and a fixed 3° anti-scatter slit on the secondary side. Axial Soller slits at 2.5° were used on both the primary and the secondary sides. The K_β_ radiation was removed with a 0.02 mm Ni-filter. The signal was collected with an LYNXEYE 1D detector with the detector stripes fully open. A corundum standard was measured with identical setup to determine the instrumental effects on the peak profile. The lattice parameters for crystalline silicon were obtained from the PDF-2015 database and verification was performed with DIFFRAC.EVA 3.1 software. TOPAS 4.2 software was used to perform a full profile Pawley fit to the measured data, where the instrumental effects determined with the corundum sample were utilized. From the fitted curve, the crystallite size was calculated from the Scherrer equation using integral breadth as the peak width, which eliminates the requirement for Scherrer constant approximation [18].

The porous structure of the NP was investigated by N_2_ physisorption using Micrometrics TriStar 3000 instrument. The sample was degassed (at 65°C in vacuum) and filled with controlled increments of N_2_ at a temperature of 77 K. The N_2_ adsorption/desorption isotherms were used to calculate the surface area using BET-theory [19], whereas single point pore volume, and pore size distribution were calculated based on the BJH-theory [20].

The surface chemistry of NP was studied with a Fourier transform infrared spectroscope (FTIR, Thermo Scientific Nicolet 8700), and the sample was prepared as tablets by mixing 250 mg KBr with 0.2 mg of the NP.

The particle size, polydispersity, and zeta potential of NP and BPQ+NP were measured at 25°C by dynamic light scattering (DLS, Zeta-sizer Nano ZS, Malvern Instruments, UK). Samples were washed thrice with Milli-Q water and resuspended in 2 mL water, and the measurements were done both before and after the freeze-drying.

### Nanoparticle internalization into macrophages

Mouse RAW 264.7 macrophages were used to study the cellular uptake of NP. The macrophages were grown in DMEM supplemented with 10% FBS, 1% penicillin/streptomycin, and 1% L-glutamine. The cells were maintained at 37°C, 95% air, and 5% CO_2_ atmosphere by changing the culture medium every second day and the cells were divided after reaching 80–90% confluency.

The intracellular distribution of NP in the macrophages was visualized by using transmission electron microscope (TEM, Jeol JEM 2100F). The cells were plated in a 12-well plate (Corning) (50 000/cm^2^), and the NP (10 μg/mL) were incubated for 1, 4, and 24 h. Cells were washed twice with Dulbecco’s Phosphate Buffered Saline (DPBS, Bio Whittaker) and fixed with 2.5% glutaraldehyde buffer solution (0.1 M phosphate buffer at pH 7.4) for 2 h and in 1% osmium buffer solution (0.1 M phosphate buffer at pH 7.4) for 1 h. The cells were washed between and after the fixations two times with the same buffer. Subsequently, the cells were dehydrated using EtOH gradient and embedded with EPON epoxy resin (LX-112). The EPON was polymerized at 60°C for 2–3 days, and approximately 70 nm thick slices were cut from the casts afterward. The sample slices were stained with 1% uranyl acetate for 30 min and 1% lead citrate for 2 min. The sample slices were placed on standard 200 mesh copper grids and imaged using TEM with an acceleration voltage of 200 kV.

### Parasites and culture conditions

*L*. *donovani* AG83 (MHOM/IN/83/AG83) and GE1 (MHOM/IN/80/GE1F8R) promastigotes were routinely grown in medium 199 supplemented with 10% heat-inactivated fetal bovine serum, 2 mM L-glutamine, 20 mM HEPES, 100 U/mL penicillin, and 100 μg/mL streptomycin at 25°C. *L*. *donovani* amastigote forms were grown and maintained as described previously [21]. Axenic amastigote forms were derived from promastigote forms by culturing them in MMA/20 (medium for axenic amastigote) at pH 5.5 [22]. Axenically grown amastigotes were maintained by sub-culture of 10^5^ parasites/ml repeated every fifth day under experimental conditions of 37°C temperature and 5% CO_2_ environment [21,23].

### Generation of drug-resistant *L*. *donovani* amastigote strains

SSG and PMM resistant *L*. *donovani* amastigotes were generated by using the methods described previously [24]. Briefly, the wild-type AG83 promastigotes were cultured in medium 199 (with supplements), in the presence of drug concentration corresponding to the 50% inhibitory concentration of the drugs (IC_50_ values) against the parasite, which was 3.6 μg/mL and 10 μM for SSG and PMM, respectively. The cultures were stabilized for three subcultures. Then, the drug concentration was increased until the cell population decreased approximately to 20% for each batch. Finally, when 90% cell population of the initial count was reduced, the phenotype generated was plated on medium 199 agar plates containing the same drug concentration, and a single colony was picked for culture in medium 199 liquid media at the same drug concentration [25,26]. The cultured promastigotes are converted into amastigotes as described above. Drug pressure was withdrawn, and stability was checked at one, two, and four months. SSG and PMM resistant phenotype clones were labeled as AG83-R,SSG and AG83-R, PMM, respectively.

### Resident peritoneal macrophage isolation

Resident peritoneal macrophages were isolated from BALB/c mice. For the isolation, 1.5 mL of sterile thioglycolate medium was injected into the peritoneal cavity to induce non-infectious inflammation. Animals were sacrificed after 5 days, and peritoneal exudates were harvested by intraperitoneal lavage. Cold RPMI (6 mL) was injected into the peritoneal cavity. After 10 min, the injected fluid was withdrawn by the same syringe. From each animal, approximately 3–4 mL of medium was recovered. After centrifugation at 200 g for 15 min, macrophages were collected. Hemocytometer was used to count the total number of isolated macrophages, and it was found that approximately 84% of total isolated cells were macrophages, as calculated by using the method previously reported [27].

### Monitoring the viability of amastigotes and macrophages

Before starting the drug sensitivity assays, the viabilities of macrophages, amastigotes and promastigotes were checked with the Trypan blue exclusion method in order to verify that the cells and the parasites were good for the experiments [28]. The number of the viable cells was verified by their intact cell membranes that excluded the dye (Trypan blue), whereas dead cells did not. The experimental protocol was such that the cell suspension of 100 μL was mixed with 100 μL of 0.4% trypan blue in phosphate buffer saline at pH 7.2 for macrophages and promastigotes, and pH 5.5 for amastigotes. The mixture was incubated for 2 min at room temperature. The sample was loaded into a hemocytometer and examined immediately under a light microscope. The number of blue staining cells were counted. The viability was more than 95% for all types of cells.

### Evaluation of antileishmanial activities against axenic amastigotes

The antileishmanial activity of the drug specimens (BPQ+NP, BPQ, SSG, AmB, PMM, and MF) against the axenic amastigotes were evaluated based on the IC_50_ value, which is the concentration of the specimen required to kill 50% of the parasites. The parasites were first seeded into 96-well plates at a density of 1x10^5^ amastigotes/well in 200 μL medium. Then, 10 μL of the drug candidates at various concentrations were added and the final volume was adjusted to 300 μL medium. The number of amastigotes in the wells treated with each specimen were compared to those in the untreated control wells.

### Cytotoxicity studies

The cytotoxicity of the drug specimen was evaluated by the CC_50_ value, which is the concentration required to kill 50% of the host cell population. Macrophages were first isolated from peritoneal lavage as described above. The cells were seeded into 96-well plates at 1x10^6^ cells/mL in 200 μL of RPMI-1640 supplemented with 10% FCS, 20 mM L-glutamine, 16 mM NaHCO_3_, penicillin (50 U/mL) and streptomycin (50 μg/ mL) at 37°C in 5% CO_2_ for 4 h for adherence. To remove the non-adherent cells, each plate was washed two times with phosphate buffer saline (pH 7.2). Plates were treated with 2-fold serial dilutions of each drug specimen. After 48 h of exposure at 37°C in 5% CO_2,_ the viable macrophages were counted microscopically using a hemocytometer.

### Evaluation of antileishmanial activity against intracellular amastigotes

The intracellular antileishmanial activity was evaluated based on the reduction in the amastigote numbers in the infected macrophages after the treatment. The method was adapted from our previous work [24]. Briefly, 4x10^6^ amastigotes in 500 μL of RPMI-1640 suspension were added to each well containing macrophages with the macrophage: parasite ratio of 1:10. The plates were incubated for 4 h at 37°C in 5% CO_2_. After the parasite internalization period, cells were washed at least twice with fresh media to remove non-phagocytosed parasites. Then, the drug specimen suspended in 1 mL RPMI-1640 was added, and the samples were incubated for 72 h at 37°C in 5% CO_2_. The medium was aspirated, the coverslips were removed, and then fixed with methanol and air-dried. After staining with Giemsa, 100 cells on the glass discs were counted. Three independent experiments in quadruplicate for each concentration were performed.

### Determination of IC_50_ and CC_50_ values

Microscopic examinations were performed in duplicates with various concentrations of pure drugs or buparvaquone-loaded nanoparticles and also for control wells. The amastigotes cells were counted for all cases using a hemocytometer. The mean of the control wells was considered as 100% survival. The counts in the treated wells at various concentrations were converted into percentages. Giemsa-stained slides of the macrophages infected with amastigotes were observed under a light microscope. Then the numbers of amastigotes and infected macrophages per 100 cells were counted for each slide. The percentage (%) of inhibition was calculated by using the equation

$$\%\;of\;inhibition=\frac{C-T}{C}\times 100,$$ (2)

where C is the number of amastigotes/infected macrophages per 100 cells in the control well and T is the number of amastigotes/infected macrophages per 100 cells in the drug-treated well.

Origin version 6.1 software was used to calculate IC_50_ and CC_50_ values by plotting a graph of the percentage of inhibition at various concentrations of all the observations.

### Resistance index and selectivity index

Resistance index (RI) is calculated using the ratio of the IC_50_ against drug resistant strain to the IC_50_ against wild-type strain (AG83). The lower RI value indicates higher efficiency against resistant strains. The selectivity index (SI) is the ratio between the cytotoxicity and the anti-parasitic activity of a drug (SI = CC_50_/IC_50_). The higher the SI value, the safer and more effective the drug is during the *in vivo* treatment. It is considered that a drug specimen presents a promising activity when the SI value is ≥10 [29].

### Antileishmanial activity *in vivo*; mice infection and treatment

#### Leishman–Donovan Unit (LDU) assay

BALB/c mice were infected through the lateral tail vein with 2×10^7^ amastigotes of wild-type AG83 *L*. *donovani* isolated from infected mice spleen. To examine the potency of infection, spleen biopsies were performed by randomly selecting 3–4 mice from each group on day 30 post-infection. Spleen smears were stained with Giemsa to confirm infection. The infected animals were divided into three groups consisting 4–5 animals per group. The groups were classified as the infected control group, BPQ treated group and BPQ+NP treated group. On the next day, the treatment was initiated by intraperitoneal administration of 0.2 mL of BPQ or BPQ+NP at a dose of 1 mg/kg body weight for 5 consecutive days. The infected control group was injected an equal volume of PBS during the entire dosing period. On day 7 post-treatment, the weight of the spleen was measured immediately after autopsy. After that spleen impression smears were prepared on glass slides and stained with Giemsa to analyze infected visceral organ (spleen) microscopically. The parasite burdens were determined by evaluating the LDU values, which were calculated by multiplying the number of amastigotes counted per 500 nuclei with the spleen weight in mg [30]. The % of amastigote inhibition was calculated using the equation

$$\%\;of\;amastigote\;inhibition=\frac{\left(NB-NA\right)}{NB}\times 100$$ (3)

and the % of amastigote replication suppression was calculated by using the equation

$$\%\;of\;amastigote\;replication\;suppression=\frac{\left(NC-NA\right)}{NC}\times 100$$ (4)

where NB is the number of amastigotes per 500 nuclei in the spleen before treatment, NA is the number of amastigotes per 500 nuclei in the spleen after treatment, and NC is the number of amastigotes per 500 nuclei in the spleen after treatment in the control group.

#### Culture microtitration assay

Three animal groups consisting 4–5 mice per group were divided as the infected control group, BPQ treated group, and BPQ+NP treated group. Mice were infected with wild-type *L*. *donovani* AG83 parasites by injecting 0.2 mL sterile PBS containing 2×10^7^ amastigotes cells in the lateral tail vein. After 30 days of infection, the infected mice were administered with 0.2 mL of sterile BPQ or BPQ+NP samples through the intraperitoneal route with a dose of 2 mg/kg at a 5-days interval. The infected control group was injected with 0.2 mL of sterile water during the entire dosing period. Seven days post-treatment, the animals were sacrificed, and their spleens were aseptically removed and weighed for determination of amastigote burden by microtitration assay as described elsewhere [31]. Wells were examined for viable and motile promastigotes. The final titer was the last dilution for which the well contained at least one parasite. The number of amastigotes per gram of spleen tissue (amastigote burden) was calculated by the following equation

$$Amastigote\;burden=\frac{Geometric\;mean\;of\;reciprocal\;titers\;from\;each\;duplicate}{Weight\;of\;homogenized\;cross\;section}\times 400.$$ (5).

### Evaluation of *in vivo* toxicity by biochemical markers

BALB/c mice were grouped into three categories containing 5 animals in each group; normal group, infected group and BPQ+NP treated group. Infection was established as described above. The BPQ+NP treated group received 4 doses of 0.2 mL of the drug loaded nanoparticles, intraperitoneally with each dose of 2 mg/kg at 5-days interval. Normal group and infected group received 0.2 mL of water for injection by similar way. After the completion of dosing schedule, blood samples were collected by cardiac puncture. Then the levels of different enzymes like alanine aminotransferase (ALT), aspartate aminotransferase (AST), blood urea nitrogen (BUN) and creatinine (CRE), were analyzed using commercially available biomarker enzymatic assay kit (Thermo Fischer Scientific).

### Statistical analysis

GraphPad Prism 5 version was used for statistical analysis. An unpaired t-test (two-tailed) was applied for the determination of significance of IC_50_ and CC_50_ values of BPQ+NP over BPQ in axenic amastigotes, intracellular amastigotes, inhibition of infected and non-infected macrophages. It was also used to analyze amastigote inhibition in spleen and amastigote replication suppression. The Mann–Whitney test (two-tailed) was used for the determination of significance of mean amastigotes per 500 nuclei in spleen and LDU values.

## Results and discussions

### Characterization of the nanoparticles and cellular uptake

Phytoliths, or biogenic porous SiO_2_ particles, are accumulated in significant amounts in many plants, including rice, horsetail, and barley. Here, phytoliths from barley husks, produced in high volumes as agricultural residues, were used to produce the silicon nanoparticles. The porous SiO_2_ powder was purified from barley husk ash and reduced to PSi by magnesiothermic reduction [15]. The material was thermally hydrocarbonized (THCPSi) to produce a hydrophobic surface enabling efficient loading of BPQ and slow release. The hydrocarbon surface was further functionalized with undecylenic acid (UnTHCPSi) to make the particles negatively charged.

**Fig 2:**
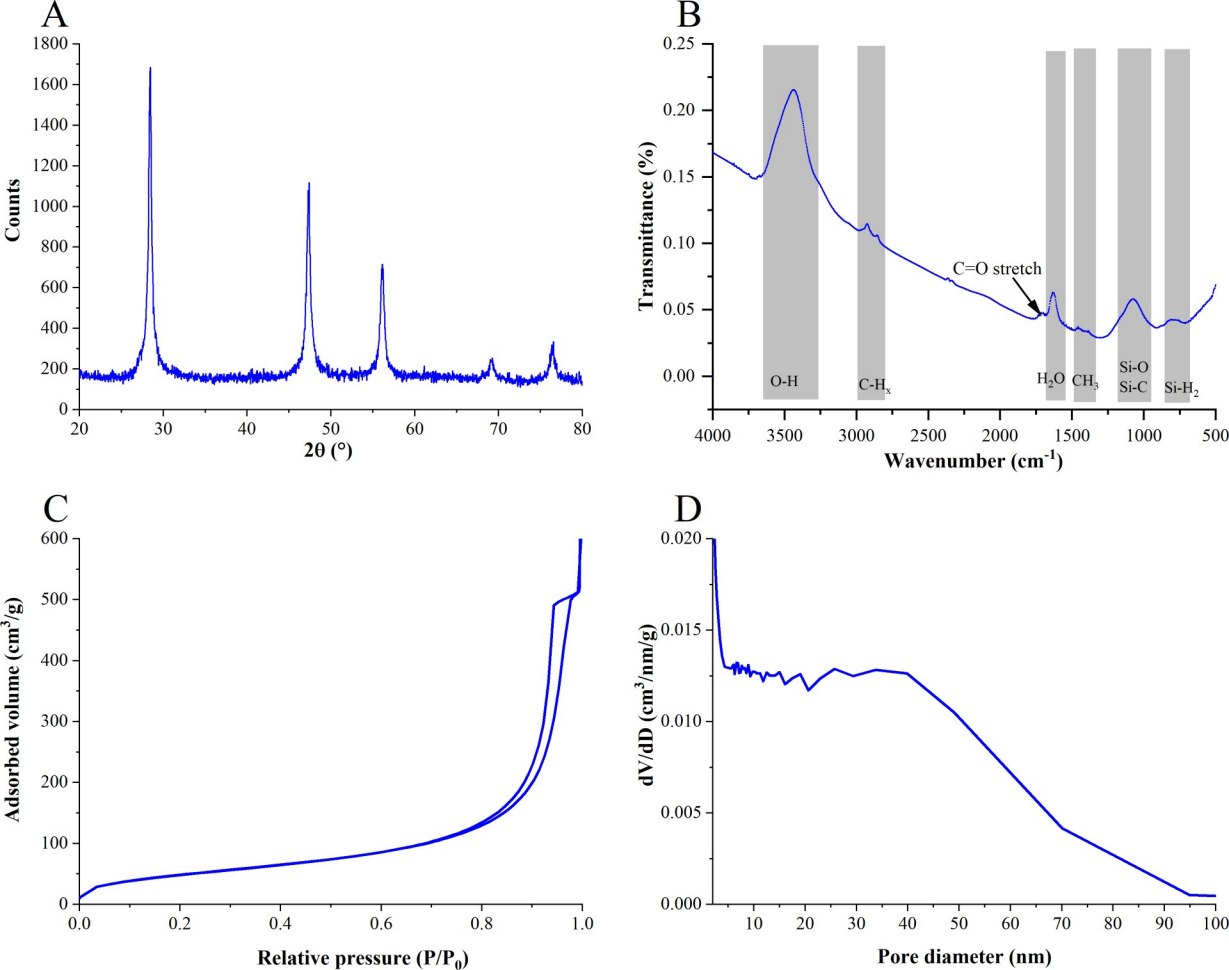
Characterization of the nanoparticles. (A) XRD diffractogram. (B) FTIR spectra. (C) N_2_ adsorption/desorption isotherms. (D) pore size distribution determined from the adsorption branch of the isotherm using the BJH theory.

The composition and crystallite size of UnTHCPSi (NP) were measured using XRD. The diffractogram consisted of broad Si peaks indicating line broadening caused by a small crystallite size of (11.4 ± 0.2) nm (Fig 2A). No other diffraction peaks were observed. In the FTIR spectra (Fig 2B), typical Si-OH groups at 3500 cm^-1^ was observed. The peaks around 2850–2970 cm^-1^ was ascertained to be the C-H_x_ groups. Characteristic stretching of C = O at 1714 cm^-1^ revealed the conjugation of carboxylic acid groups. The peak at 1630 cm^-1^ was attributable to the O−H bending of H_2_O molecules adsorbed on the sample surfaces. Peak around 1100 cm^-1^ was assigned to Si−O−Si stretching and around 600 cm^-1^ to Si-H_2_ terminations.

The pore structure was characterized by N_2_ sorption (Fig 2C). The NP had a high specific surface area of 183 ± 3 m^2^/g characteristic to nanoporous materials. The pore volume was 0.793 ± 0.006 cm^3^/g, and the pore size distribution was wide, consisting mainly of pores < 100 nm in diameter, the majority of them being < 40 nm (Fig 2D). It should be noted that the sample consisted of both inter and intraparticle pores and therefore the pore volume and pore size distribution are not directly indicative of the properties of the dispersed particles.

The loading degree of BPQ in the nanoparticles was 7% w/w. After the loading, the hydrodynamic size of the nanoparticles slightly increased (ca. 10%), but it did not affect the surface charge, as measured by DLS (Table 1). The increase in the particle size can be attributed to the adsorption of BPQ molecules on the surface of the nanoparticles and slight aggregation of small particles. The measured polydispersity index (PDI) values of all samples were between 0.1–0.2, indicating monodispersed nanoparticle suspension.

**Table 1:** Results of DLS measurements of the nanoparticles before and after freeze-drying (mean ± σ, n = 3).

Samples	Before freeze-drying			After freeze-drying		
	nm^a^	PDI^b^	mV^c^	nm^a^	PDI^b^	mV^c^
UnTHCPSi (NP)	135 ± 1	0.105 ± 0.009	- 24.3 ± 0.2	128 ± 1	0.119 ± 0.006	- 18.6 ± 0.7
BPQ+NP	147 ± 2	0.124 ± 0.016	- 24.4 ± 1.1	140 ± 2	0.167 ± 0.025	- 19.2 ± 0.6

^a^ Hydrodynamic size of the nanoparticles.

^b^ Polydispersity index

^c^ Zeta-potential of the nanoparticles

Even after the freeze-drying there were no essential differences in the particle sizes, PDI values, nor the zeta potentials of the nanoparticles. Also, the visual inspection did not show agglomeration. The results affirmed that the characteristics of the nanoparticles were well-preserved, and freeze-drying facilitated the long-term storage of the nanoparticles. Thus, the freeze-dried nanoparticles were utilized for further investigations.

**Fig 3:**
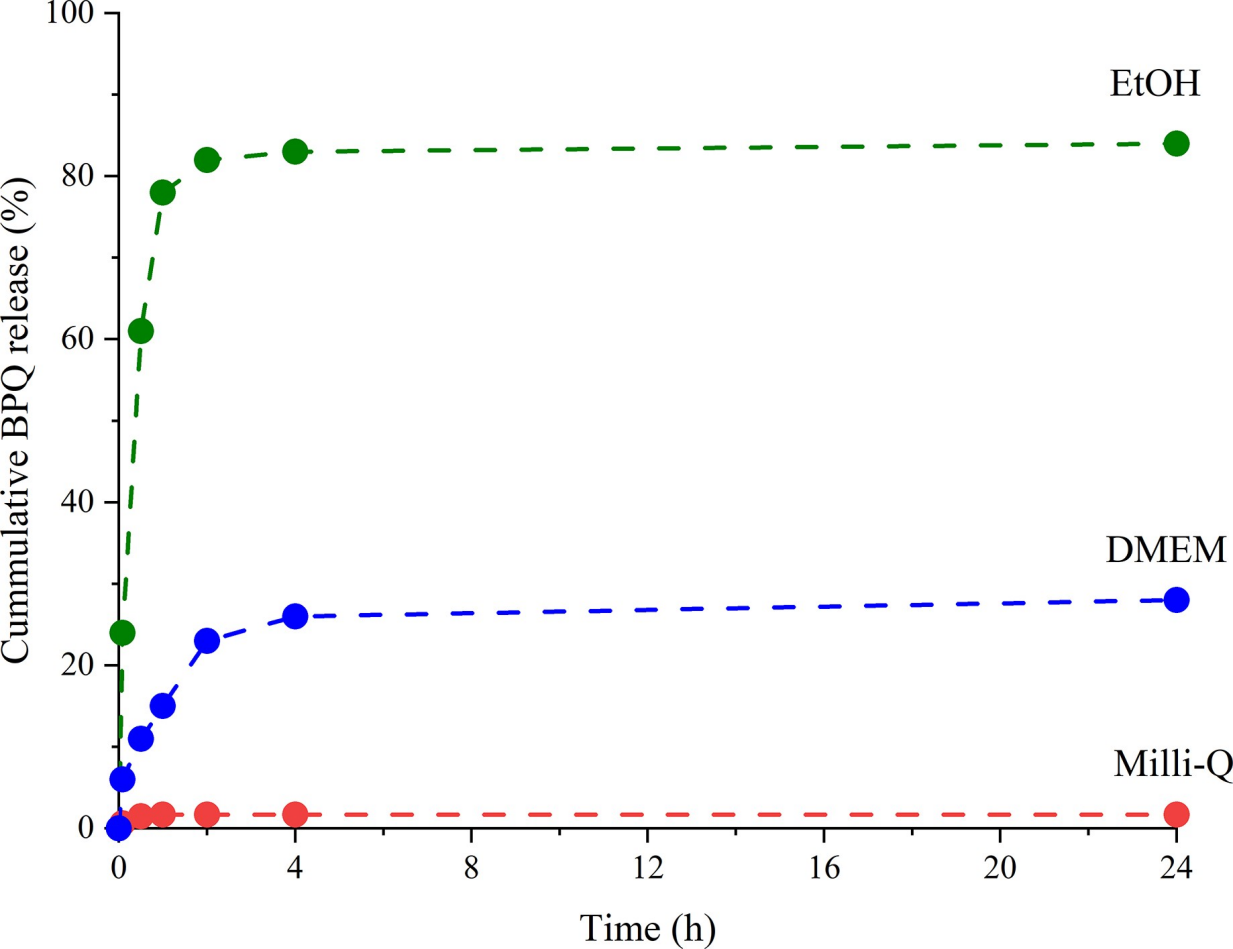
Release kinetics of BPQ from the freeze-dried BPQ+NP samples in different media.

**Fig 4:**
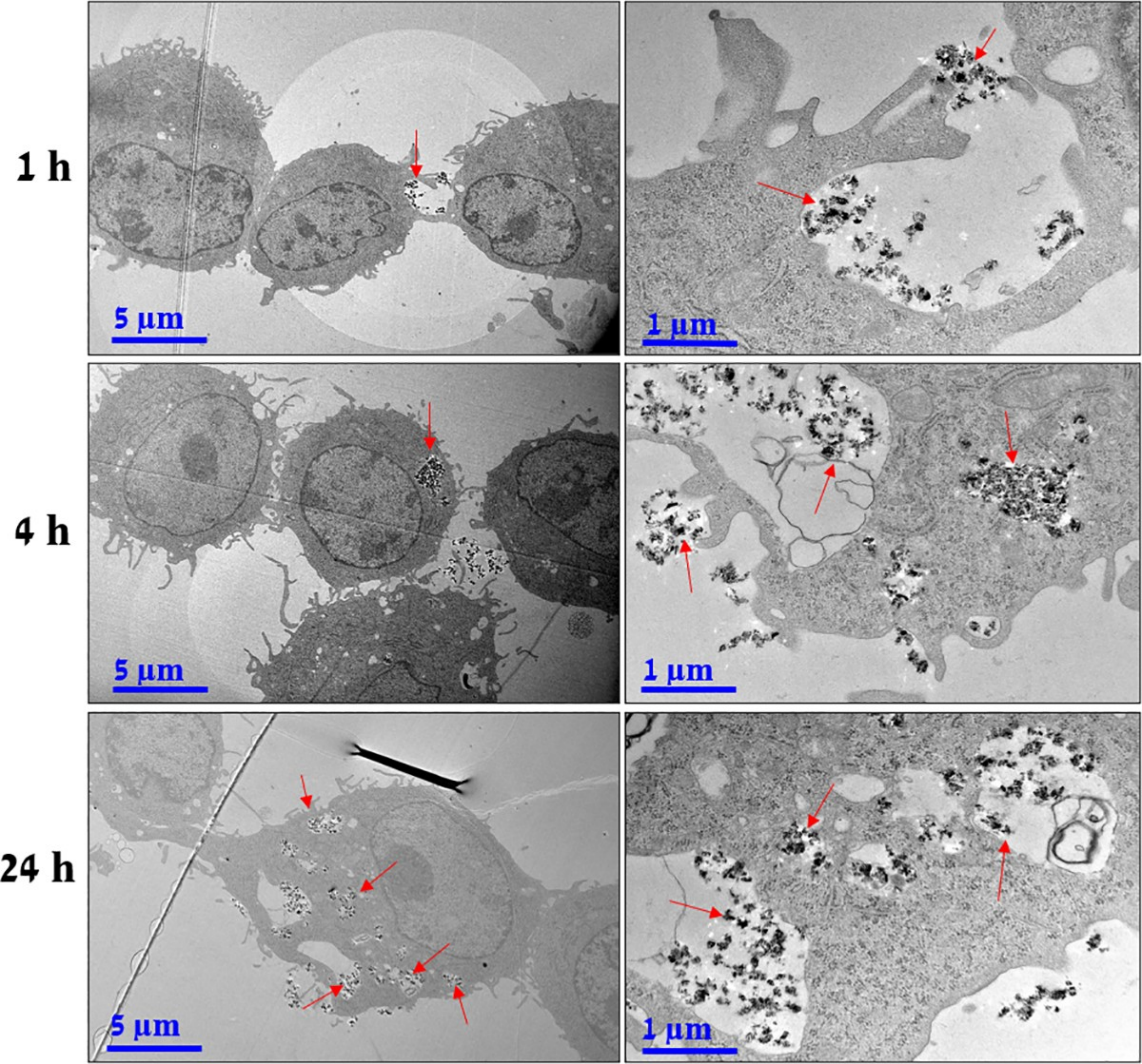
TEM images showing uptake of NP (indicated by red arrows) in mouse RAW 264.7 macrophages after 1 h (upper row), 4h (middle row), and 24 h (bottom row).

*In vitro* release kinetics of BPQ (Fig 3) showed that nearly 80% of BPQ was released in EtOH within 1 h from the freeze-dried nanoparticles. By contrast, the release in water was negligible (< 2%) due to the hydrophobic nature of the drug even in 24 h. The release in cell culture medium (DMEM, pH 7.4) was reasonably low (< 10% in 1 h and < 30% in 24 h), implying that the majority of BPQ is still loaded inside the nanoporous structure of the carrier during phagocytosis into the macrophages. The cellular internalization process via phagocytosis started already at 1 h through the accumulation of the nanoparticles on the cell membranes (Fig 4). The internalization is seen within 4 h and the number of the nanoparticles increased inside the cells up to 24 h.

### Anti-leishmanial activity against axenic and intracellular amastigotes

The efficacies of the drug specimens (BPQ+NP, BPQ, SSG, AmB, PMM, and MF) were examined against four different strains of *L*. *donovani* amastigotes both in the axenic and the intracellular model. Two main strains of the amastigotes were used: AG83 and GE1. The AG83 is a wild-type strain sensitive to SSG, whereas GE1 is SSG resistant isolate. The wild-type AG83 strain was further generated into SSG resistant (AG83-R, SSG) and PMM resistant (AG83-R, PMM) strains to broaden the spectrum of anti-leishmanial activity of the drug specimen.

In the axenic model, the anti-leishmanial activity of BPQ was essentially improved when loaded into the nanoparticles (BPQ+NP). The activity was measured by the IC_50_ values, which were higher with pure BPQ in comparison to BPQ+NP against all types of *L*. *donovani* strains (Table 2). This means that high dose of pure BPQ was required to achieve the same inhibitory effect as with BPQ+NP. The concentrations of pure BPQ were higher than the BPQ+NP by 3, 8, 7 and 4-fold against the wild-type AG83, (AG83-R, SSG), (AG83-R, PMM), and GE1 respectively. Nevertheless, the IC_50_ values with BPQ+NP were not as low as with AmB and MF, regardless of the type or the strain of *L*.*donovani*. But the efficiency of BPQ+NP in terms of the RI values were comparable with AmB and MF, in which low RI value indicated high efficiency of the drug against the amastigotes.

**Table 2:** Drug sensitivity profiles against axenic *L*.donovani amastigotes (mean ± σ, n = 4).

Drug	Axenic IC_50_ (μM)^a^				Resistance index (RI)^b^		
	Wild-type AG83^c^	AG83-R, SSG^d^	AG83-R, PMM^e^	GE1^f^	AG83-R, SSG	AG83-R, PMM	GE1
BPQ+NP	0.9 ± 0.2*	1.2 ± 0.2*	1.1 ± 0.2*	0.9 ± 0.2*	1.2 ± 0.2*	1.1 ± 0.3*	1.1 ± 0.2*
BPQ	3.4 ± 0.3**	8.1 ± 0.8*	7.1 ± 0.8*	4.1 ± 0.8*	2.4 ± 1.2*	2.1 ± 1.5*	1.2 ± 1.4*
SSG^g^	3.5 ± 0.7	133 ± 15	113 ± 15	14 ± 2	38 ± 4	32 ± 3	4 ± 1
AmB	0.2 ± 0.1*	0.3 ± 0.1*	0.3 ± 0.1*	0.3 ± 0.1*	1.1 ± 0.1*	1.2 ± 0.2*	1.1 ± 0.2*
PMM	12 ± 1*	384 ± 23*	327 ± 12*	25 ± 2*	32 ± 3**	27 ± 2**	2.1 ± 0.3*
MF	0.5 ± 0.1*	0.6 ± 0.1*	0.5 ± 0.1*	0.5 ± 0.1*	1.3 ± 0.2*	1.1 ± 0.2*	1.1 ± 0.3*

^a^ Drug concentration required to kill 50% of the amastigotes, calculated using Eq (2).

^b^ IC_50_ against drug resistant strain / IC_50_ against wild-type strain (AG83).

^c^ Wild-type *L*.*donovani* amastigotes.

^d^ SSG resistant *L*.*donovani* strain generated from AG83.

^e^ PMM resistant *L*.*donovani* strain generated from AG83.

^f^ Isolated *L*.*donovani* strain resistant to SSG.

^g^ Values for SSG were given in μg/ml.

* p < 0.001, significant difference compared with SSG.

** p > 0.05, no significant difference compared with SSG.

In the cellular model, pure BPQ reached the CC_50_ values over 300 μM and IC_50_ values at the range of only 2–5 μM (Table 3). These values are consistent with the values of >229.75 in peritoneal macrophages and 1.5 μM against *L*. *infantum* infection for CC_50_ and IC_50_, respectively, reported by Reimao *et al*. [32]. These high CC_50_ values suggest that the BPQ has only little *in vitro* toxicity to the healthy macrophages, whereas the small IC_50_ values indicate the high activity in killing the intracellular amastigotes. In case with BPQ+NP, the CC_50_ values were even larger than with pure BPQ, implying that the BPQ+NP was less toxic to the host cells (Table 3). This means that the nanoparticles are functional carriers to deliver BPQ inside the cells, and most of the drug is consumed in killing of the intracellular amastigotes. Like in axenic model, the IC_50_ values with BPQ+NP was lower than pure BPQ indicating that the BPQ+NP was more active than the pure BPQ to kill the amastigotes. The IC_50_ values of pure BPQ were larger than with BPQ+NP by 3, 5, 5, and 4-fold against AG83, (AG83-R, SSG), (AG83-R, PMM), and GE1 strain, respectively. The efficacy to selectively kill the *L*. *donovani* amastigotes were assessed by the selectivity index (SI); the higher the SI value, the more selective the drug was to kill the amastigotes while the cellular toxicity was diminished. According to the SI values, BPQ+NP were clearly more selective in comparison with pure BPQ as well as other conventional drugs against both the wild-type and the drug-resistant strains. It should be noticed in Tables 2 and 3 that there was cross-resistances of (AG83-R, PMM) to SSG and (AG83-R, SSG) to PMM.

**Table 3:** Drug sensitivity profiles against intracellular *L*.donovani amastigotes (mean ± σ, n = 4).

Drug	CC_50_ (μM)^a^	Cellular IC_50_ (μM)				Selectivity index (SI)^b^			
		Wild-type AG83	AG83-R, SSG	AG83-R, PMM	GE1	AG83	AG83-R, SSG	AG83-R, PMM	GE1
BPQ+NP	1080 ± 90*	0.9 ± 0.2*	1.1 ± 0.1*	0.9 ± 0.1*	0.9 ± 0.1*	1190 ± 70*	1050 ± 50*	1100 ± 50*	1140 ± 40*
BPQ	330 ± 30*	2.9 ± 0.3**	5.4 ± 0.4*	4.8 ± 0.5*	3.8 ± 0.4*	120 ± 10*	60 ± 10*	60 ± 10*	90 ± 10*
SSG^c^	27 ± 2	1.8 ± 0.2	22 ± 2	20 ± 2	7.5 ± 0.3	15 ± 4	1 ± 1	2 ± 1	4 ± 2
AmB	15 ± 2**	0.2 ± 0.1*	0.2 ± 0.1*	0.3 ± 0.1*	0.2 ± 0.1*	80 ± 10*	70 ± 10*	70 ± 10*	90 ± 10*
PMM	260 ± 20*	9 ± 2*	140 ± 10*	120 ± 10*	20 ± 3*	30 ± 5*	2 ± 1**	2 ± 1**	13 ± 2*
MF	36 ± 4**	0.4 ± 0.1*	0.7 ± 0.1*	0.5 ± 0.5*	0.5 ± 0.1*	100 ± 10*	60 ± 5*	70 ± 10*	80 ± 10*

^a^ Drug concentration required to kill 50% of host cell population (mean ± σ, n = 3)

^b^ CC_50_/IC_50_.

^c^ Values for SSG were given in μg/ml

* p < 0.001, significant difference compared with SSG.

** p > 0.05, no significant difference compared with SSG.

### Treatment efficacy on *in vivo* intracellular amastigotes

#### LDU assay

In LDU experiment spleens were taken for determination of organ weight and parasite burden to the immediate effects of drugs and nanoformulation on intracellular amastigotes (Table 4). The mice were treated for 5 consecutive days (1 mg/kg). Parasite burden *in vivo* was assessed by microscopic observation of amastigotes against host cell nuclei on tissue imprints and expressed as LDU values. The amastigote burden in spleen in the groups treated with BPQ and BPQ+BP was low. The amastigote burden in spleen was reduced more effectively by 20-fold and 170-fold in the BPQ and BPQ+NP treated groups, respectively when compared with the control group. Crucially, the inhibition of the amastigotes with BPQ+NP reached nearly 100% while with pure BPQ, it was only around 50%. Nevertheless, the amastigote replication suppression by BPQ was over 90% either with or without the use of the nanoparticles.

**Table 4:** *In vivo* responses to amastigotes in the spleen of infected mice using LDU assay (mean ± σ, n = 5).

Group	Spleen wt. (mg)	Amastigotes per 500 nuclei	LDU (× 10^4^)^a^	% of amastigote inhibition^b^	% of amastigote replication suppression^c^
Before treatment	130 ± 10	410 ± 60			
Infected control	180 ± 10	2580 ± 610	45 ± 13		
BPQ treated	100 ± 10	200 ± 20	2 ± 1	54 ± 13	93 ± 1
BPQ+NP treated	80 ± 10	27 ± 3	0.3 ± 0.1*	94 ± 4*	99 ± 1*

^a^ LDU (Leishman Donovan Unit) = number of amastigotes per 500 nuclei × spleen weight in mg.

^b^ Calculated using Eq (3)

^c^ Calculated using Eq (4)

* p **<** 0.01, significant compared to BPQ.

#### Culture microtitration assay (CMA)

Using the quantification of the amastigotes by simple microscopic observation may lack some amastigotes in stained organ. We carried out more sensitive culture microtitration assay (CMA) to evaluate the parasite burden by determining the number of live amastigotes present in the infected spleen. The mice were dosed either 2 or 4 times at 5 days interval (2 mg/kg). After seven days post-treatment the number of amastigotes per gram of spleen was calculated using CMA method. With free BPQ, 50% of the parasite burden was reduced, whereas the BPQ+NP removed the burden almost by 100% (Table 5). The number of dosing strengthen the effect both with BPQ (52% vs. 64%) and with BPQ+NP (89% vs. 98%). These numbers are in concordance with the results in LDU method indicating that BPQ+NP was more effective than BPQ in the VL therapy in mice.

**Table 5:** *In vivo* antileishmanial activity of BPQ and BPQ+NP on infected mice using CMA method (mean ± σ, n = 5).

Groups	No. of dosing^a^	Spleen (mg)	Suppression (%)^b^
Uninfected control		106 ± 16	
Infected control		146 ± 24	
Treated with BPQ	2	140 ± 22	52 ± 3*
Treated with BPQ	4	131 ± 18	64 ± 4*
Treated with BPQ+NP	2	119 ± 16	89 ± 4*
Treated with BPQ+NP	4	110 ± 17	98 ± 1*

^a^ Dose by intraperitoneal (i.p) injection (mg/kg body weight) after every 5 days.

^b^ Suppression of *L*. *donovani* amastigote burden in spleen calculated by using Eq 4.

* p < 0.0001, extremely significant.

### *In vivo* toxicity

**Fig 5:**
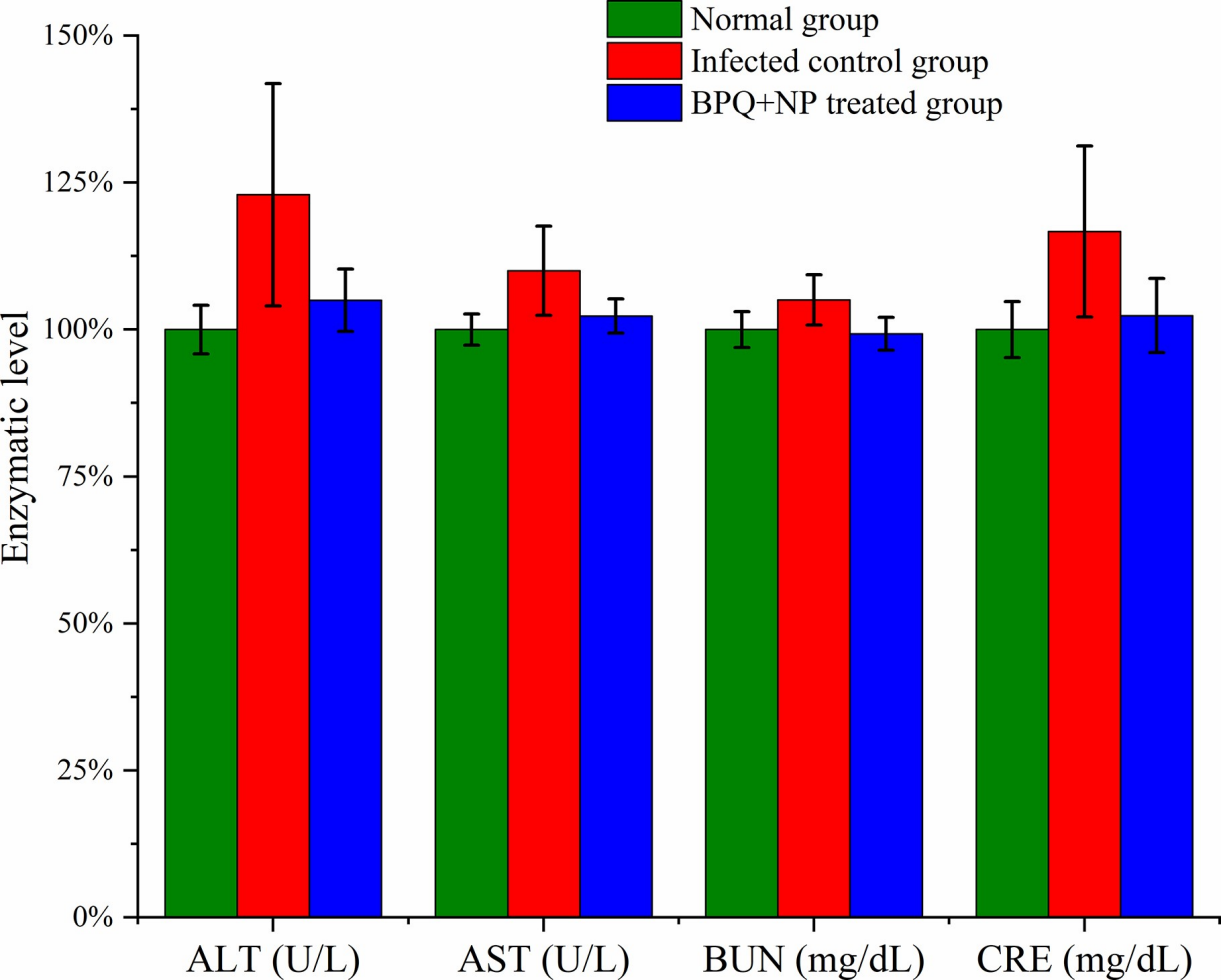
Evaluation of *in vivo* toxicity of BPQ+NP by biochemical markers (mean ± σ, n = 3). The differences between the values are not statistically significant (p > 0.05).

The levels of biological markers, alanine aminotransferase (ALT), aspartate aminotransferase (AST), blood urea nitrogen (BUN), and creatinine (CRE) were analyzed to study the *in vivo* toxicity to liver and kidney (Fig 5). The biomarker levels of the normal mice were considered 100%, to which the toxicity levels of the infected mice and the BPQ+NP group were compared. With the normal mice infected with *L*. *donovani*, the biomarker levels increased even though the differences are not statistically different (p > 0.05). However, all four biochemical markers remained at the normal level with the BPQ+NP group. The results indicate that BPQ+NP were not toxic in repeated dosing, and there was no kidney or liver injury.

### Significances of the nanocarriers and possible mechanism of action

Ever since the anti-leishmanial activity of BPQ was discovered, new strategies to improve its bioavailability has been introduced. For example, derivation of more water soluble BPQ prodrugs (buparvaquone-3-phosphate and 3-phosphonooxymethyl-buparvaquone) were developed [12]. The buparvaquone-3-phosphate was shown to be the most effective prodrug but only small reduction (34%) in the liver parasite burden in BALB/c mice was achieved [12,33].

Apart from BPQ, new therapeutics such as bisnaphthalimidopropyl derivatives [34] and plant-based extracts e.g., artemisinin [35] have been shown as potential anti-leishmanial drugs but their utility in native form is limited because of their low bioavailability and high toxicity. However, when the drugs were incorporated with nanoparticles, their efficacies were significantly improved [34,35]. For example, artemisinin loaded with poly(_D,L_-lactic-*co*-glycolic acid) PLGA nanoparticles reduced the parasite burden infected with *L*.*donovani* to 85% (liver) and 82% (spleen) while the free drug reduced the burdens only moderately (~50%) [35]. Similarly, Lima *et al*., reported the anti-leishmanial activity of bisnaphthalimidopropyl loaded PLGA nanoparticles to be 80% (spleen) and up to 50% (liver) more effective than the free form of the drug against *L*. *infantum* infections [34]. As the nanoparticles themselves were non-toxic, the activities of the payloads were significantly elevated to combat the VL infections.

Despite the therapeutics, such as BPQ, being nonresistant to VL, the toxicity hinders their use in native form and requires a delivery platform to make them safer and improve their bioavailability and efficacy. Drug formulations based on functionalized nanocarrier is an option to facilitate their therapeutic use. For active drug targeting, the nanoparticle surfaces have been anchored with a variety of specific moieties like mannose [36–38] and phosphatidylserine (PS) [32,39–42]. The crafted functional moiety operated as active ligands that specifically bind with the receptor of infected cells, and therefore, enhanced the selectivity and uptake of the nanocarriers towards the target site. For example, mannose anchored nanoparticles were more effective to transport AmB to the diseased site in comparison with the bare nanoparticles or pure AmB [36–38]. *In vivo* studies examined by Shahnaz *et al*., showed that the efficacy of AmB loaded in mannose grafted thiolated chitosan nanoparticles was as high as 89%, while the non-targeted thiolated chitosan nanoparticles showed only 36% reduction in the *L*.*donovani* burden in spleen [36]. Similarly, doxorubicin loaded in PS grafted nanocapsules inhibited the parasite burden by 85% in spleen whereas the efficacy of doxorubicin was lower (73%) when loaded with unfunctionalized nanocapsules [42]. Another study conducted by Khatik *et al*., revealed that AmB loaded in PS modified gelatin nanoparticles reduced the parasite burden infected with *L*. *donovani* in spleen (85%) whereas with bare gelatin nanoparticles, the efficacy reduced to 71% at corresponding doses of AmB [41].

In case with BPQ, when the drug was encapsulated with PS grafted liposomes, the efficacy of BPQ was substantially improved. For instance, the parasite burden in the spleen and in the liver were reduced up to 98 and 96%, respectively [39]. The improved efficacy was attributed to greater interaction of negatively charged PS-liposomes with “scavenger receptors” of the macrophages leading to effective internalization of the encapsulated BPQ [32,39,40].

Even though surface functionalization has shown to improve the targeting efficacy of the nanocarriers, their wider use is meager due to the complex processes involved in the grafting of the functional component [43]. Consequently, the production cost of the nanomedicine is susceptible to escalate. Herein, we produced affordable biogenic PSi nanocarriers from low-cost agricultural residue (barley husk), alongside valorizing the biomass. Conventionally, PSi nanoparticles are produced by electrochemical etching of Si wafers, but their production cost is relatively high and may not be viable in VL therapy since the disease is mostly endemic in developing countries. Furthermore, biocompatibility of PSi in drug delivery systems has been explored in several biomedical applications. The PSi is biodegradable material that does not induce toxicity at moderate concentrations since the degradation product, orthosilicic acid, is non-toxic to human body, which is eventually excreted through the urine [44–47].

In the present study, the biogenic PSi have been shown to be biocompatible since the cytotoxicity level of BPQ were significantly lowered when encapsulated in nanoparticles (BPQ+NP) in comparison with pure BPQ and other conventionally used drugs. The *in vitro* results revealed that the BPQ-loaded nanoparticles were effectively internalized into the cells, and thereafter, enhanced the permeability of BPQ inside the cells. Subsequently, the BPQ-loaded in the nanoparticles had reasonable anti-leishmanial activity both against the wild-type and the drug resistant *L*. *donovani* strains. *In vivo* inhibition studies examined in spleen also revealed that the efficacy of BPQ was significantly improved when the drug was encapsulated in the biogenic PSi nanoparticles. The parasite burden was almost completely inhibited with BPQ+NP (~100%), while the inhibition was only 50% with the native BPQ.

For leishmaniasis chemotherapy trypanothione system, surface lipids, polyamine and sterol biosynthesis, and efflux pumps are putative drug targets [48,49]. In addition, P-glycoprotein in the infected cells plays major role for multidrug resistance which limits the leishmaniasis chemotherapy. Nanotechnology based drug delivery systems have the ability to overcome drug resistance and improve cellular uptake due to the nano size of the particles which makes them capable to penetrate through biological barriers, enhance cellular uptake and deliver the drug in infected tissues [50–52]. Furthermore, it has been well established that the nanoparticles overcome multidrug resistance by down-regulating the overexpression of P-glycoprotein [53,54].

It can be proclaimed that the pH-responsive coordination interactions between the hydrocarbonized surfaces of the nanoparticles and BPQ not only improved its loading efficiency but also helped in its controlled release in the acidic phagolysosomal environment. Moreover, the internalized nanocarriers typically inhibit the efflux pump proteins to remove the drug molecules from the cells making the treatment more effective alleviating the drug resistance [55]. So, after the endocytosis of BPQ+NP into the infected cells, P-glycoprotein could not pump out the nanoparticles and get rid of being effluxed. This could be the main reason for the improved antileishmanial activity of BPQ when loaded in biogenic nanocarriers against drug resistant strains of VL.

Nevertheless, specific surface functionalization of the PSi nanoparticles may further improve their selectively and uptake towards the targeted site. In future, thorough investigations of the drug loaded nanoparticles in terms of different administration routes, toxicity, and their side effects shall be studied in more detail.

## Conclusion

Visceral leishmaniasis is a neglected tropical disease for which no vaccine is available. Novel treatments against leishmaniasis are globally focused on repositioning of approved drugs. Buparvaquone has been used long in veterinary treatment against bovine theileriosis. It has been shown to have excellent *in vitro* activity against *L*. *donovani*, but its lipophilic nature hinders its use *in vivo*. Herein, we loaded the buparvaquone in biogenic porous silicon nanoparticles to improve its bioavailability. The nanoparticles were synthesized from barley husk, which is an agricultural residue and widely available. This approach to develop low-cost biogenic nanocarrier system is also an efficient way to valorize the biomass. The nanoparticles were able to encapsulate buparvaquone adequately and release it in a controlled manner intracellularly. The *in vitro* antileishmanial studies revealed that the buparvaquone-loaded nanoparticles were susceptible to the wild-type and different resistant strains of the *L*. *donovani* amastigotes with high degree of selectivity. The present work reports for the first time the successful utilization of low-cost biogenic nanomaterial in the antileishmanial nanomedicine having minimal toxicity, improved cellular uptake and high degree of selectivity against drug-resistant visceral leishmaniasis. New affordable and low-toxic multifunctional nanomedicine was developed as an alternative to treat the macrophage associated neglected tropical disease that affects mainly the poor people in the developing countries. The present study opens the route to further develop the porous silicon nanoparticles, for instance, by anchoring specific ligands on the particle surface to improve targeting. Such nanomedicine has high potential to be developed from the laboratory to the clinic, and to establish a new treatment regimen for antileishmanial chemotherapy especially in case of the drug resistant visceral leishmaniasis.

## Supporting information

S1 Scheme Semantic diagram representing the buparvaquone loaded nanoparticles (left) and their internalization in macrophage infected with visceral leishmaniasis (right).
(TIF)
