# Supplementary figures and images for: Biogenic nanoporous silicon carrier improves the efficacy of buparvaquone against resistant visceral leishmaniasis
Source: PLoS Negl Trop Dis. 2021 Jun 29;15(6):e0009533. doi: 10.1371/journal.pntd.0009533 (PMC8274846; doi:10.1371/journal.pntd.0009533)

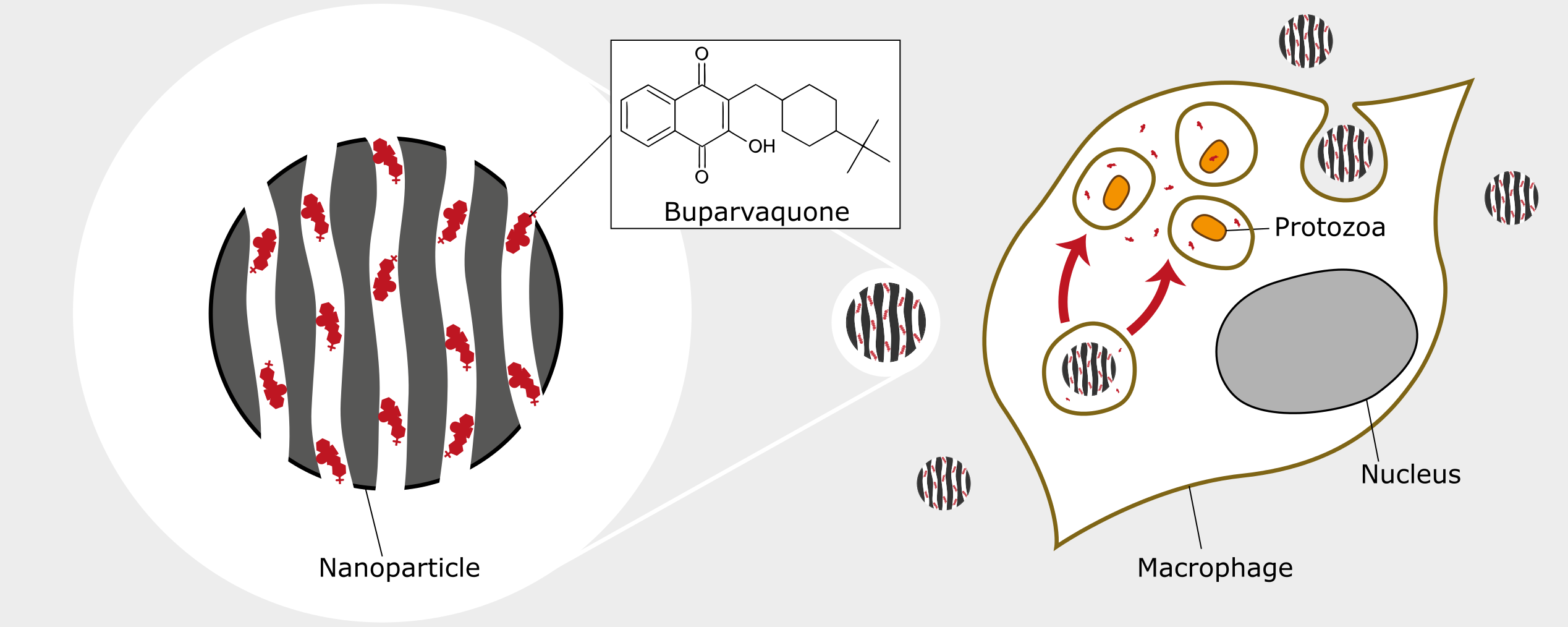

Supplement: S1 Scheme — Semantic diagram representing the buparvaquone loaded nanoparticles (left) and their internalization in macrophage infected with visceral leishmaniasis (right). (TIF) [file pntd.0009533.s001.tif]
